# O.3.2-6 A computer-tailored physical activity intervention for prostate and colorectal cancer patients and survivors: long-term efficacy and behavior change maintenance of OncoActive

**DOI:** 10.1093/eurpub/ckad133.151

**Published:** 2023-09-11

**Authors:** Rianne Henrica Johanna Golsteijn, Catherine Bolman, Denise Astrid Peels, Esmee Volders, Hein De Vries, Lilian Lechner

**Affiliations:** Open Universiteit, The Netherlands; Open Universiteit, The Netherlands; Open Universiteit, The Netherlands; Open Universiteit, The Netherlands; Maastricht University, The Netherlands; Open Universiteit, The Netherlands

## Abstract

**Purpose:**

To improve the long-term physical and psychological health of prostate and colorectal cancer survivors, physical activity (PA) interventions aimed at maintaining PA are necessary. OncoActive is a print- and web-based intervention that was was able to increase PA of prostate and colorectal cancer patients and survirors shortly after the intervention ended. This study evaluated the long-term efficacy (12-month follow-up) comparing OncoActive and a control group. Furthermore, we explored whether the increased PA was maintained after an 8 month non-intervention follow-up period.

**Methods:**

Prostate or colorectal cancer patients were randomly assigned to OncoActive (N = 249) or a usual care control group (N = 229). OncoActive participants received 3 times PA advice and a pedometer. PA outcomes (i.e., ActiGraph and self-report MVPA min/week and days with ≥ 30 min PA) and health-related outcomes (i.e., fatigue, depression, physical functioning) that were significantly changed by the intervention at 6-month follow-up, were included. Differences between groups and changes over time were assessed with multilevel linear regressions.

**Results:**

At 12 months, OncoActive participants did not perform better than control group participants at ActiGraph MVPA min/week, self-report MVPA min/week or ActiGraph days with PA. Only self-report days with PA were significantly higher in OncoActive compared to the control group. Fatigue was the only health-related outcome that was significantly lower in OncoActive compared to the control group at 12-month follow-up. When exploratively looking within the OncoActive group, the previously found PA increase shortly after the intervention (6 months follow-up) was maintained at 12 months and PA improved significantly from baseline to 12 months. The control group showed small but non-significant improvements from 6 to 12 resulting in a decline of differences between groups.

**Conclusion:**

At long-term follow-up (i.e., 12-months), most significant differences between OncoActive and the control group at short-term follow-up (i.e., 6 months) were no longer significant. Natural improvements in the control group may have contributed to this. Fatigue was significantly lower in OncoActive compared to control group participants at 12 months. Computer-tailored PA advice may give participants an early start to recovery and potentially contributes to improving long-term health.

**Funding:**

KWF Kankerbestrijding (Dutch cancer society) NOU2012-5585

